# Alterations in gut microbiome and metabolite profile of patients with *Schistosoma japonicum* infection

**DOI:** 10.1186/s13071-023-05970-3

**Published:** 2023-10-05

**Authors:** Chen Zhou, Junhui Li, Chen Guo, Zhaoqin Zhou, Zhen Yang, Yu Zhang, Jie Jiang, Yu Cai, Jie Zhou, Meng Xia, Yingzi Ming

**Affiliations:** 1 Transplantation Center, Engineering and Technology Research Center for Transplantation Medicine of National Health Commission, The Third Xiangya Hospital, Central South University, Changsha, Hunan China; 2 Schistosomiasis Control Institute of Hunan Province, Yueyang, Hunan China

**Keywords:** *Schistosoma japonicum* infection, Gut microbiome, Metabolite profile, Disease progression

## Abstract

**Background:**

Schistosoma infection is a significant public health issue, affecting over 200 million individuals and threatening 700 million people worldwide. The species prevalent in China is *Schistosoma japonicum*. Recent studies showed that both gut microbiota and metabolome are closely related to schistosomiasis caused by *S. japonicum*, but clinical study is limited and the underlying mechanism is largely unclear. This study aimed to explore alterations as well as function of gut microbiota and metabolite profile in the patients with *S. japonicum* infection.

**Methods:**

This study included 20 patients diagnosed with chronic schistosomiasis caused by *S. japonicum*, eight patients with advanced schistosomiasis caused by *S. japonicum* and 13 healthy volunteers. The fresh feces of these participators, clinical examination results and basic information were collected. 16S ribosomal RNA gene sequencing was used to investigate gut microbiota, while ultraperformance liquid chromatography-mass spectrometry (UHPLC-MS) was applied to explore the metabolome of patients in different stages of schistosomiasis.

**Results:**

The study found that gut microbiota and metabolites were altered in patients with different stages of *S. japonicum* infection. Compared with healthy control group, the gut microbial diversity in patients with chronic *S. japonicum* infection was decreased significantly. However, the diversity of gut microbiota in patients with chronic schistosomiasis was similar to that in patients with advanced schistosomiasis. Compared with uninfected people, patients with schistosomiasis showed decreased *Firmicutes* and increased *Proteobacteria*. As disease progressed, *Firmicutes* was further reduced in patients with advanced *S. japonicum infection*, while *Proteobacteria* was further increased. In addition, the most altered metabolites in patients with *S. japonicum* infection were lipids and lipid-like molecules as well as organo-heterocyclic compounds, correlated with the clinical manifestations and disease progress of schistosomiasis caused by *S. japonicum*.

**Conclusions:**

This study suggested that the gut microbiota and metabolome altered in patients in different stages of schistosomiasis, which was correlated with progression of schistosomiasis caused by *S. japonicum*. This inter-omics analysis may shed light on a better understanding of the mechanisms of the progression of *S. japonicum* infection and contribute to identifying new potential targets for the diagnosis and prognosis of *S. japonicum* infection. However, a large sample size of validation in clinic is needed, and further study is required to investigate the underlying mechanism.

**Supplementary Information:**

The online version contains supplementary material available at 10.1186/s13071-023-05970-3.

## Background

*Schistosoma*, the cause of schistosomiasis, affects over 200 million individuals and threatens 700 million people in the world [1]. Five species are responsible for human schistosomiasis, including *Schistosoma mansoni*, *S. japonicum, S. haematobium, S. intercalatum* and *S. mekongi* [2]. In China, the most prevalent species is *S. japonicum*, and schistosomiasis caused by *S. japonicum* remains a significant public health issue [3, 4].

Schistosomiasis caused by *S. japonicum* can be classified into three distinct stages based on the progression of the disease, including acute, chronic and advanced *S. japonicum* infection[5]. However, the pathophysiology of different stages of schistosomiasis caused by *S. japonicum* is complex and not well defined. Evidence suggested that the interaction between the host immune cells and worms or eggs plays a critical role in the pathogenesis of schistosomiasis caused by *S. japonicum* [6]. Recently, studies indicated that gut microbiota might influence the development of schistosomiasis [7–13]. Dysbiosis of gut microbiome was observed in *Schistosoma* infection in mouse models [8, 9, 13, 14]. Furthermore, specific changes in the gut microbiome community structure could assist early diagnosis and prognosis[7]. Animal study suggested that decreased *Roseburia* and *Ruminococcaceae* UCG-014, as well as increased *Staphylococcus*, *Alistipes* and *Parabacteroides*, indicated a higher risk of infection [7]. Although study on the role of gut microbiota in schistosomiasis draws huge interest, clinical research in this area is limited. Our previous study suggested that distinct changes in gut microbiota seemed to have potential to indicate different stages of *S. japonicum* infection and act as diagnostic biomarkers [8]. However, further validation in the clinic is required, and mechanisms need to be explored.

Metabolomics is a relatively newly developed discipline after genomics and proteomics, which explores the interaction of all metabolites in a cell or tissue at a certain time [9]. Metabolites can effectively reflect various physiological and pathological processes. Due to the high sensitivity of metabolomics, it can often detect subtler biological changes [10]. Metabolomics has been widely studied in a variety of diseases [11]. In parasitic diseases, metabolomics has also been used to reveal pathology of parasitic diseases[12]. Therefore, metabolomics has potential to provide a powerful complement for revealing the mechanisms of chronic schistosomiasis and advanced schistosomiasis and shed light on developing new approaches for diagnosis and treatment.

In this study, we investigated the alterations of the gut microbiota and metabolome in patients in different stages of schistosomiasis with 16S ribosomal RNA gene sequencing and ultraperformance liquid chromatography-mass spectrometry (UHPLC-MS). Also, we explored the correlation between the intestinal microbiota and metabolic profiles in patients with *S. japonicum* infection. This inter-omics analysis will promote a deeper understanding of the mechanisms of the progression of *S. japonicum* infection and contribute to identifying new potential targets for the diagnosis and prognosis of *S. japonicum* infection.

## Methods

### Study design

The study was carried out in the Xiang Yue Hospital in Yue Yang, Hunan Province, China, from October 2021 to October 2022. Among all the patients with *S. japonicum* infection, we included 20 patients with chronic *S. japonicum* infection, eight patients with advanced *S. japonicum* infection and 13 healthy volunteers from the same area according to strict inclusion and exclusion criteria. The study was performed in accordance with the Code of Ethics of the World Medical Association (Declaration of Helsinki) for experiments. Approval was granted by the Clinical Research Ethics Committee of the Third Xiangya Hospital of Central South University (Fast I 21145). All patients and healthy volunteers signed an informed consent form and received privacy protection.

### Inclusion criteria for patients with chronic *S. japonicum* infection

According to the National Standardized Diagnostic Criteria for *S. japonicum* infection (WS261-2006) of the Ministry of Health of China, the inclusion criteria of patients with chronic *S. japonicum* infection were as follows:

1. Living in an epidemic area or having a history of contact with epidemic water for many times.
2. Asymptomatic or intermittent abdominal pain, abdominal distension or bloody stool, enlargement of the left lobe or splenomegaly.
3. Schistosoma eggs were observed in fecal examination or serological examination was positive.
4. B-ultrasound examination showed hepatic fibrosis.

### Inclusion criteria for patients with advanced *S. japonicum* infection

According to the National Standardized Diagnostic Criteria for *S. japonicum* infection (WS261-2006) of the Ministry of Health of China, the inclusion criteria of patients with advanced *S. japonicum* infection were as follows:

1. Living in an epidemic area or having a history of contact with epidemic water for many times.
2. There are clinical manifestations of portal hypertension, colonic granuloma or dwarfism.
3. Schistosoma eggs were observed in fecal examination or serological examination was positive.
4. B-ultrasound examination showed hepatic fibrosis.

### Exclusion criteria

1. Patients with tumor.
2. Patients in acute infection, including acute respiratory infection, acute gastrointestinal infection, acute skin and soft tissue infection, acute intracranial infection, etc.
3. Patients infected with hepatitis A, B, C, D or E viruses.
4. Patients with other parasitic diseases, such as *Toxoplasma gondii*, malaria, tapeworm, *Fasciola hepatica*, etc.
5. Patient with other infectious diseases, such as tuberculosis, syphilis, AIDS.
6. Patients with diabetes, hyperthyroidism and other underlying diseases.
7. Patients with cardiovascular disease.

The basic information of the participants is summarized in Table 1.

**Table 1:** Basic information of study population per group

Variables	H	CS	AS	Difference
n	13	20	8	
Age(years)	55 ± 3.24	53 ± 2.30	56 ± 3.65	0.4207
Gender(male/female)	7/6	10/10	4/4	0.9741
Active smoker (%)	23.1	30.0	25.0	0.9006
Active drinker (%)	15.4	15.0	12.5	0.9816
Dietary type	Eastern diet	Eastern diet	Eastern diet	None
	100%	100%	100%	

There were no statistically significant differences among the three groups. ANOVA was used to evaluate whether age has an effect on the experiment, F = 0.8858, R^2^ = 0.1111. Differences in gender, active smoker and drinker were determined using the Chi-square test, *χ*^2^ of gender = 0.05256, *df* = 2, *χ*^2^ of smoker = 0.2093, *df* = 2, *χ*^2^ of drinker = 0.0371, *df* = 2. *H* Healthy people, *CS* chronic *Schistosoma japonicum* infection, *AS* advanced *S. japonicum* infection

### Sample collection

All subjects received professional training before sample collection. The fresh stool samples were put into a sterile collection tube within half an hour, quickly frozen in liquid nitrogen for 20 min and then transferred to a cryotube and stored in a refrigerator at – 80 ℃. Two fecal samples from the same patient were kept, one for gut microbiota research and one for metabolomics study.

### 16sRNA sequencing

#### Extraction of genome DNA

Genomic DNA extraction of fecal samples was carried out by a Magnetic Soil and Stool DNA Kit (DP712, Tiangen), according to the manufacturer's instructions. Sample, buffer and grinding beads were added to the centrifuge tube and vortexed and the supernatant saved according to the manufacturer's instructions. We added buffer SH, buffer GFA and magnetic bead suspension to the supernatant successively, shook them and mixed them well. The centrifuge tube was placed on a magnetic stand for protein removal, rinsing, elution and other operations to finally obtain a DNA solution according to the manufacturer's instructions. The DNA concentration was detected by NanoDrop (Thermo Scientific), while the integrity and purity of DNA were assessed by 1.0% agarose gel electrophoresis. DNA was diluted with sterile water to a concentration of 1 ng/μl.

#### Amplicon generation

Phusion High-Fidelity PCR Master Mix (15 μl) (M0531L, New England Biolabs) was used to perform the PCR reaction. The primers 341F (5′-CCTACGGGNGGCWGCAG-3′) and 806R (5′-GGACTACHVGGGTATCTAAT-3′) were applied to amplify the V3–V4 regions of the 16S rRNA gene. Primers, Phusion High-Fidelity PCR Master Mix and template DNA were thoroughly mixed and then put in a PCR machine. Thermal cycling program included initial denaturation at a temperature of 98 ℃ for 60 s, then 30 cycles of denaturation at a temperature of 98 ℃ for 10 s, followed by annealing at a temperature of 50 ℃ for 30 s, and next elongation at a temperature of 72 ℃ for 60 s, then keeping it at 72 ℃ for 5 min.

#### PCR product quantification and qualification

The PCR products and 1 × loading buffer with SYB green were mixed well at the ratio of 1: 1, and 2.0% agarose gel electrophoresis was performed to analyze the PCR products. The DNA within bright main bands between 400 and 450 bp were selected for subsequent experiments.

#### PCR product mixing and purification

Add 5 volumes of buffer PB to 1 volume of the PCR product sample and then mix according to the manufacturer's instructions. Prepare the vacuum manifold and QIA quick columns. Close unused positions with luer caps. To bind DNA, load the samples into the QIA quick columns by decanting or pipetting. To wash, add 0.75 ml buffer PE to each QIA quick column and apply a vacuum. Transfer each QIA quick column to a microcentrifuge tube or the provided 2-ml collection tubes. Centrifuge for 1 min at 17,900 × g. To elute DNA, add 50 µl of buffer EB. Put elution buffer in the center of each QIA quick membrane, let the columns stand for 1 min and then centrifuge.

#### Library preparation and sequencing

The sequencing libraries were generated by a NEB Next® Ultra™ DNA Library Prep Kit for Illumina (E7370L, NEB), and barcodes were added according to the manufacturer's instructions. The library quality was analyzed on the Qubit@ 2.0 Fluorometer (Thermo Scientific) and Agilent Bioanalyzer 2100 system (Agilent Technologies). Then, sequencing of the library was carried out on an Illumina NovaSeq6000 to generate 250-bp paired-end reads.

#### 16sRNA data analysis

Merging paired-end reads from the original DNA fragments were performed by FLASH (http://ccb.jhu.edu/software/FLASH/). Sequence analysis was carried out with UPARSE software package (http://drive5.com/uparse/). Alpha and beta diversity was analyzed by using in-house Perl scripts. Choose 97% as the similarity criterion and sequences with similarity > 97% will be assigned to the same OTUs. A representative sequence for each OTU was picked, and its taxonomic information was annotated by RDP classifier. QIIME was used to explore alpha diversity, while LEfSe (http://huttenhower.sph.harvard.edu/lefse/) was applied for the quantitative analysis. ANOSIM was carried out based on the Bray-Curtis dissimilarity distance matrices to detect differences in gut microbial communities among different groups.

### Untargeted metabonomic

#### Sample preparation

Samples were quick-frozen in liquid nitrogen immediately and ground into fine powder. Add 1000 μl methanol/acetonitrile/H2O (2:2:1, v/v/v) to homogenized solution for subsequent metabolites extraction. The solution was centrifuged for 20 min at 14,000 g at 4 °C. The solution supernatant was dried via vacuum centrifuge. To perform LC-MS analysis, 100 μl acetonitrile/water (1:1, v/v) solvent was added to the samples to re-dissolve it, and then it was centrifuged for 15 min at 14,000 g at 4 ℃; finally, the supernatant was collected.

#### UHPLC-Q-exactive orbitrap MS

Analysis was carried out by an UHPLC (Vanquish UHPLC, Thermo) coupled to an Orbitrap (Q Exactive HF-X/Q Exactive HF, Thermo) at Shanghai Applied Protein Technology Co., Ltd. To perform HILIC separation, samples were processed with a 2.1 mm × 100 mm ACQUIY UHPLC BEH Amide 1.7 µm column (Waters, Ireland).The mobile phase contained A = 25 mM ammonium acetate and 25 mM ammonium hydroxide in water and B = acetonitrile in both ESI-positive and -negative modes. The gradient was 98% B for 1.5 min and then linearly reduced to 2% for 10.5 min, then was maintained for 2 min and next increased to 98% for 0.1 min, following a 3-min re-equilibration period. The ESI source condition parameters were as follows: Ion Source Gas1 (Gas1) as 60; Ion Source Gas2(Gas2) as 60; curtain gas (CUR) as 30; source temperature: 600 ℃; IonSpray Voltage Floating (ISVF) ± 5500 V. In MS-only acquisition, the equipment was set to acquire over the m/z range 80–1200 Da, the resolution was turned to 60,000, and the accumulation time was turned to 100 ms. In auto MS/MS acquisition, the equipment was regulated to acquire over the m/z range 70–1200 Da, the resolution was adjusted to 30,000, and the accumulation time was adjusted to 50 ms, excluding time within 4 s.

#### Data processing

Before being imported into XCMS software, the raw MS data were converted to MzXML files via Proteo Wizard MS Convert. The parameters used for peak picking were as follows: cent Wave m/z = 10 ppm, peakwidth = c (10, 60), prefilter = c (10, 100). The following parameters were used for peak grouping: bw = 5, mzwid = 0.025, min frac = 0.5. Annotation of isotopes and adducts was performed by CAMERA (Collection of Algorithms of MEtabolite pRofile Annotation). In the extracted ion features, the variables with > 50% of the nonzero measurement values were selected and used for further analysis. Metabolite compound identification was carried out by analyzing of accuracy m/z value (< 10 ppm), and MS/MS spectra with an in-house database were established with authentic standards.

#### Statistical analysis

The sum-normalized processed data analysis was performed with R package (ropls). The processed data received multivariate data analysis, including Pareto-scaled principal component analysis (PCA) as well as orthogonal partial least square discriminant analysis (OPLS-DA). The robustness of the model was assessed by both sevenfold cross-validation and response permutation testing. In the OPLS-DA model, the variable importance in the projection (VIP) value of all variables was calculated to assess its classification contribution. Differences between two groups with independent samples were analyzed by Student’s t test. Metabolites with VIP > 1 and *p* value < 0.05 were recognized to be significantly changed. The correlation between two variables was determined by Pearson’s correlation analysis. Spearman correlation hierarchical cluster analysis was used to study the relationship between the gut microbiota and metabolites.

## Result

### Composition of gut microbiota in patients with *S. japonicum* infection at different stages

**Fig. 1:**
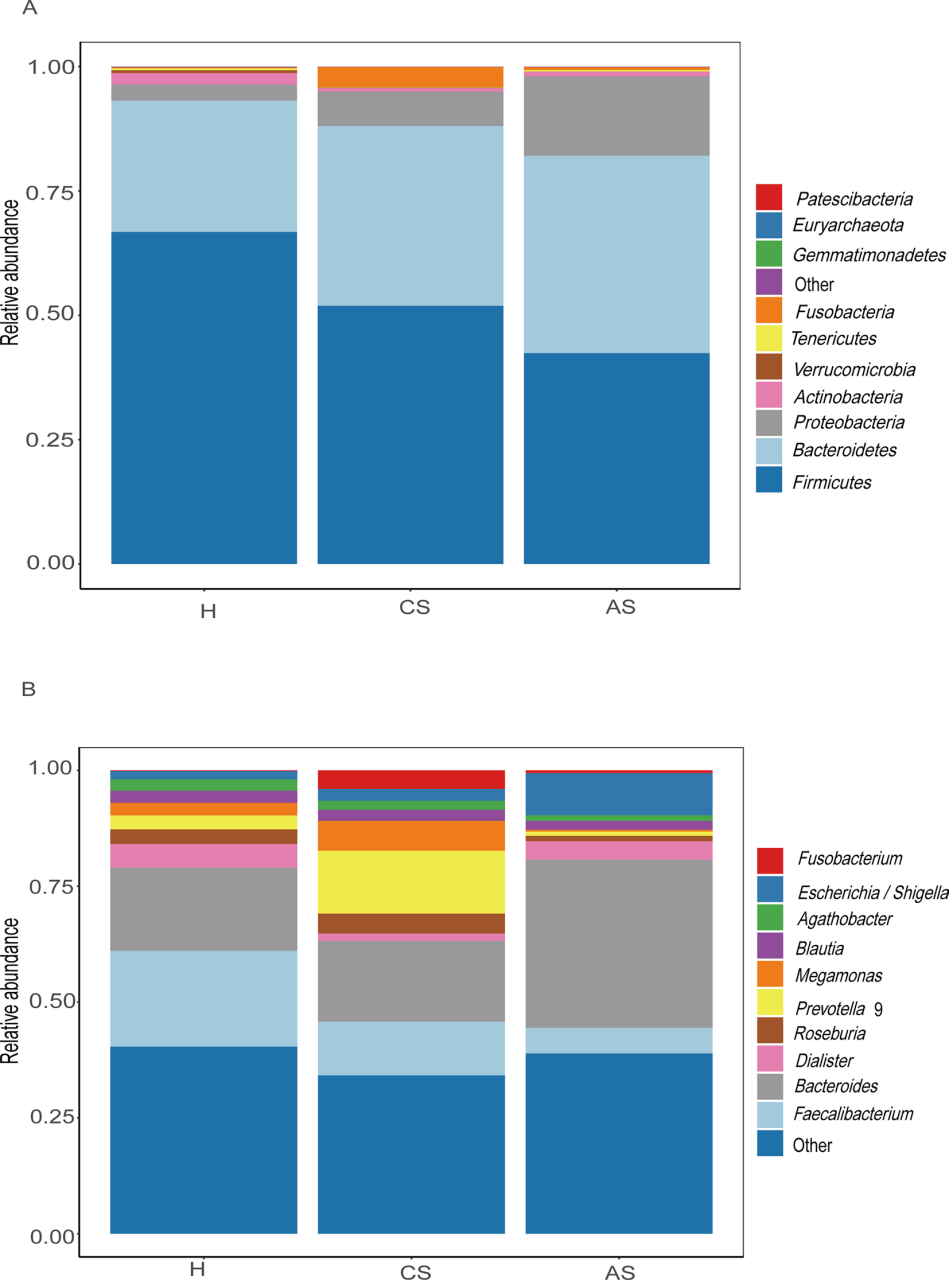
Community structure of the gut microbiota of per groups. **A** Phylum level. **B** Genus level. *H* healthy people, *CS* chronic *S. japonicum* infection, *AS* advanced *Schistosoma japonicum* infection

To explore the changes of the intestinal flora in patients with *S. japonicum* infection, we investigated the community structure of the gut microbiota of patients with chronic *S. japonicum* infection, patients with advanced *S. japonicum* infection and healthy volunteers. At the phylum level (Fig. 1A), *Firmicutes, Bacteroidetes* and *Proteobacteria* were the top three most abundant gut microbiomes in all groups. Compared with uninfected people, patients with *S. japonicum* infection showed decreased *Firmicutes* and increased *Proteobacteria*. As the disease progressed, *Firmicutes* in patients with advanced *S. japonicum* infection was further reduced, while *Proteobacteria* was further increased. Additionally, we investigated the alterations at the genus level (Fig. 1B). We found that *Faecalibacterium* and *Bacteroides* were the top two most abundant gut microbes. When the patient was infected with *S. japonicum* and as the disease progressed, the amount of *Faecalibacterium* decreased. As patients with chronic *S. japonicum* infection progressed to advanced stages, *Bacteroides* gradually became the dominant bacteria. There were also bacteria that did not show consistent trends in patient disease progression. For example, the content of *Dialister* was reduced in the gut of patients with chronic *S. japonicum* infection compared with healthy people, but in advanced stages, its content recovered. In addition, the contents of *Prevotella* 9 and *Megamonas* gradually increased after the patients were infected with *S. japonicum*, but decreased in the patients with advanced *S. japonicum* infection. Notably, compared with healthy people, patients with advanced *S. japonicum* infection have a significantly higher level of *Echerichia/Shigella*. Our results suggested that patients with *S. japonicum* infection at different stages have unique intestinal microbial communities.

### Alterations in diversity of patients infected with *S. japonicum*

**Fig. 2:**
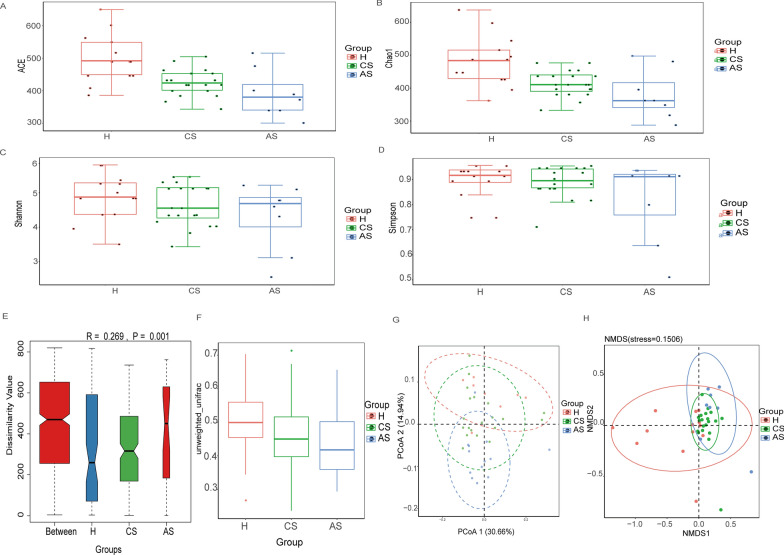
Alpha and beta diversity of the three groups. **A** Ace index(H vs CS:t-test,* t*_(*31*) _= 2.45,  *P = 0.0058*; H vs AS: t-test, *t*_(*19*) _= 2.53, *P = 0.0054*; CS vs AS:*P = 0.2379*). **B** Chao1 index(H vs CS: t-test, *t*_(*31*)_ = *2.45*, *P = 0.0055*; H vs AS: t-test, *t*_(*19*)_ = 2.53, *P = 0.0095*; CS vs AS:*P = 0.0055*). **C** Shannon index(H vs CS: *P = 0.3628*; CS vs AS:*P = 0.5326*). **D** Simpson index(H vs CS:*P = 0.8702*; CS vs AS:*P = 0.3284*). **E** ANOSIM analysis (*R = 0.269*, *P = 0.001*). **F** Unweighted Unifrac beta analysis(H vs CS:t-test, *t*_(*31*)_ =3.63 *P<0.0001*; CS vs AS:*P = 0.5466)* . **G** PcoA. **H** NMDS. *H* healthy people, *CS* chronic *Schistosoma japonicum* infection, *AS* advanced *S. japonicum* infection. *PcoA* principal coordinates analysis, *NMDS* non-metric multidimensional scaling

After initially exploring the community structure of gut microbes, we then analyzed the diversity of gut microbes in the three groups. Four statistical methods were applied to compare the alpha diversity of the three groups. The Ace index (Fig. 2A) and chao1 index (Fig. 2B) suggested that alpha diversity of gut microbiota was significantly reduced in patients with chronic *S. japonicum* infection compared with healthy individuals (Ace: t-test, *t*_*(31)*_ = 2.45,  *P* = *0.0058*; Chao1: t-test, *t*_*(31)*_ = 2.45, *P* = *0.0055*), whereas the Shannon index (Fig. 2C) and Simpson index (Fig. 2D) showed no difference between the two groups (Shannon: *P* = 0.3628; Simpson: *P* = 0.8702). For patients with advanced *S. japonicum* infection, the alpha diversity was also reduced compared with healthy people (Ace: t-test, *t*_*(19)*_ = 2.53, *P* = *0.0054*; Chao1: t-test, *t*_*(19)*_ = 2.53, *P* = *0.0095*). Patients with chronic *S. japonicum* infection and patients with advanced *S. japonicum* infection did not show significant changes (Ace: *P* = 0.2379; Chao1: t-test, *t(26)* = 2.48, *P* = *0.0055*; Shannon: *P* = 0.5326; Simpson: *P* = 0.3284). To further compare the species diversity of gut microbiota among the healthy, chronic and advanced groups, we performed a beta diversity analysis. ANOSIM analysis showed that the difference among the three groups was greater than the difference within the group (*R* = 0.269, *P* = 0.001), indicating that the grouping in this study was reasonable and the statistical results were credible (Fig. 2E). Compared with healthy people, the beta diversity of gut microbiota was significantly reduced in patients with chronic *S. japonicum* infection (t-test, *t(31)* = 3.63, *P* < 0.0001), but there was no significant difference between patients with chronic *S. japonicum* infection and patients with advanced *S. japonicum* infection (*P* = 0.5466) based on unweighted Unifrac beta (Fig. 2F). To observe the differences in beta diversity of the three groups more intuitively, we conducted PcoA (Fig. 2G) and NMDS analysis (Fig. 2H). They showed that there were differences between patients with chronic *S. japonicum* infection and healthy people. Patients with advanced *S. japonicum* infection showed greater variability compared with healthy people. There was no significant difference between patients with chronic *S. japonicum* infection and those with advanced *S. japonicum* infection. This result was consistent with unweighted Unifrac beta analysis. Thus, alpha and beta diversity of gut microbiota was significantly lower in patients with chronic *S. japonicum* infection compared with healthy individuals, but the diversity of gut microbiota was similar between patients with chronic *S. japonicum* infection and patients with advanced *S. japonicum* infection.

### Species analysis of differences among groups

**Fig. 3:**
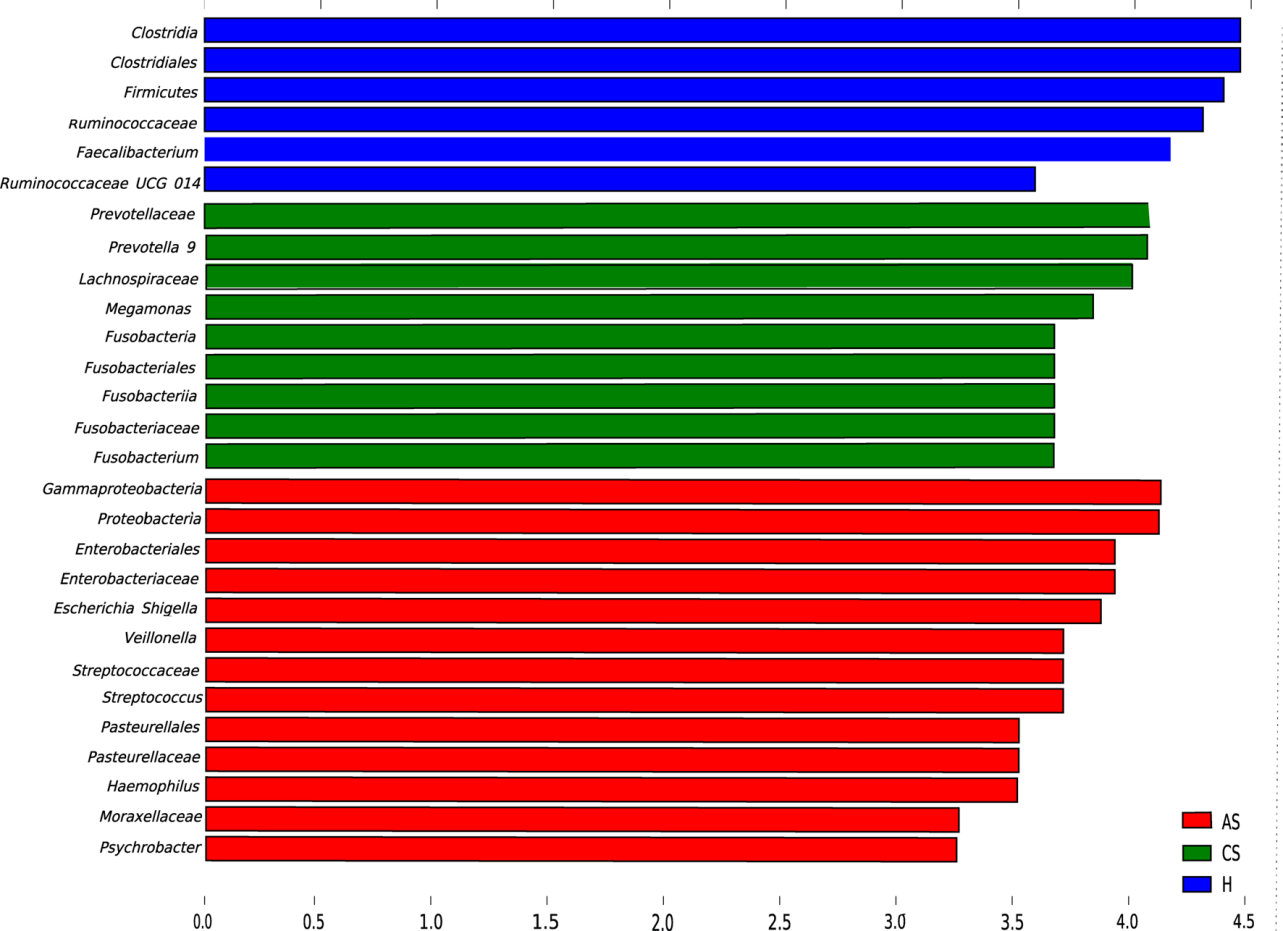
LEfSe analysis (LDA score > 2) to explore the different intestinal flora in the three groups. *H* healthy people, *CS* chronic *Schistosoma japonicum* infection, *AS* advanced *S. japonicum* infection, *LDA* linear discriminant analysis

LEfSe analysis was performed to analyze intestinal microbial species in each group. LDA > 2 was used as screening criteria, and we found 39 species abundant in normal people, 15 species specific in patients with chronic *S. japonicum* infection and 34 species abundant in patients with advanced *S. japonicum* infection (Fig. 3). *Clostridia, Clostridiales, Firmicutes, Ruminococcacea* and *Faecalibacterium* were significantly associated with healthy people (LDA score > 6). The intestinal tract of patients with chronic *S. japonicum* infection contained a large number of *Prevotellaceae* and *Prevotella* 9 (LDA score > 6), while *Gammaproteobacteria* and *Proteobacteria* became the characteristic strains in patients with advanced *S. japonicum* infection (LDA score > 6).

### Function prediction

**Fig. 4:**
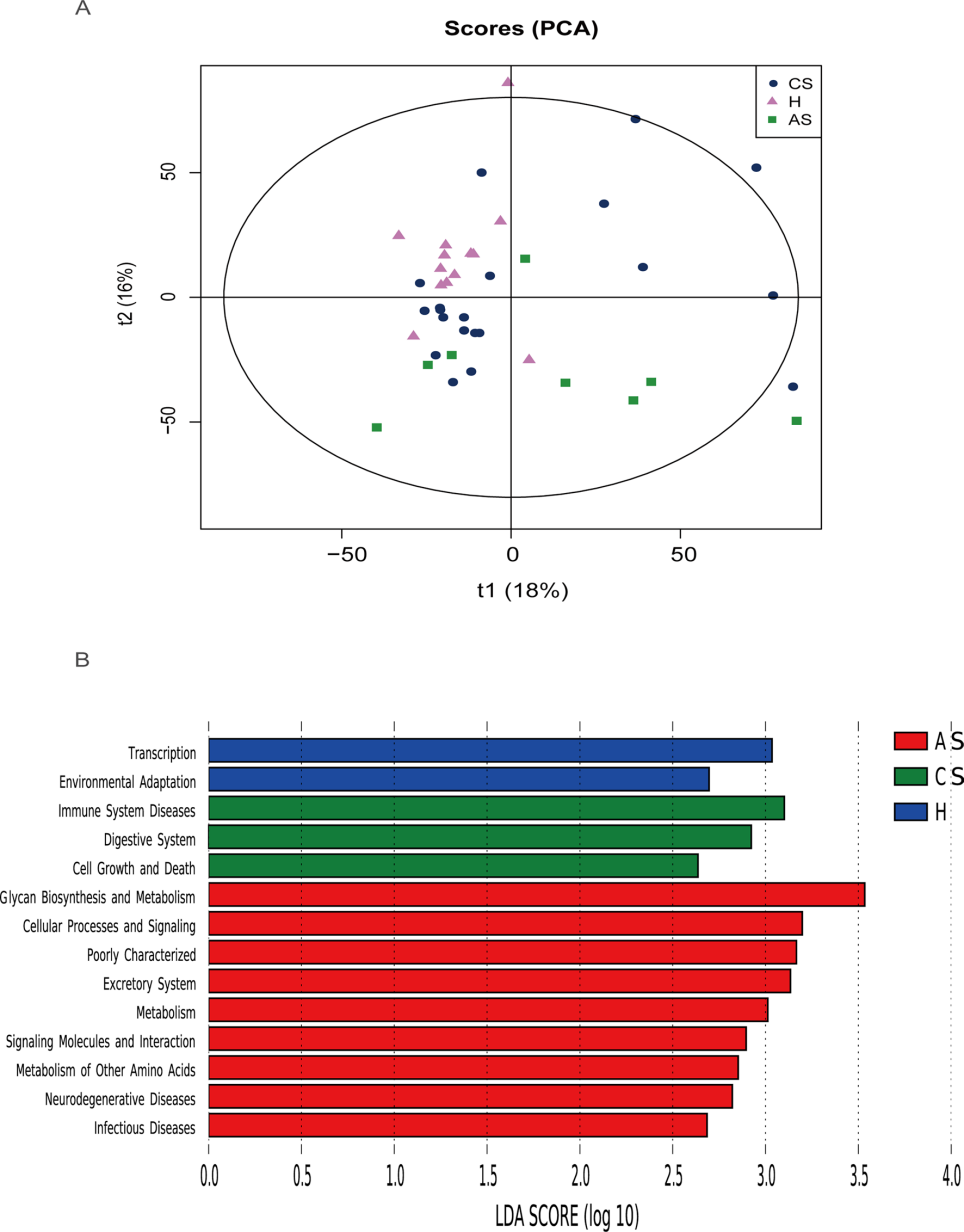
Function prediction of three groups. **A** PCA analysis based on KEGG database. **B** LEfSe analysis of the three groups (LDA score > 2). *H* healthy people, *CS* chronic *Schistosoma japonicum* infection, *AS* advanced *S. japonicum* infection, *LDA* linear discriminant analysis. *KEGG* Kyoto Encyclopedia of Genes and Genomes

**Fig. 5:**
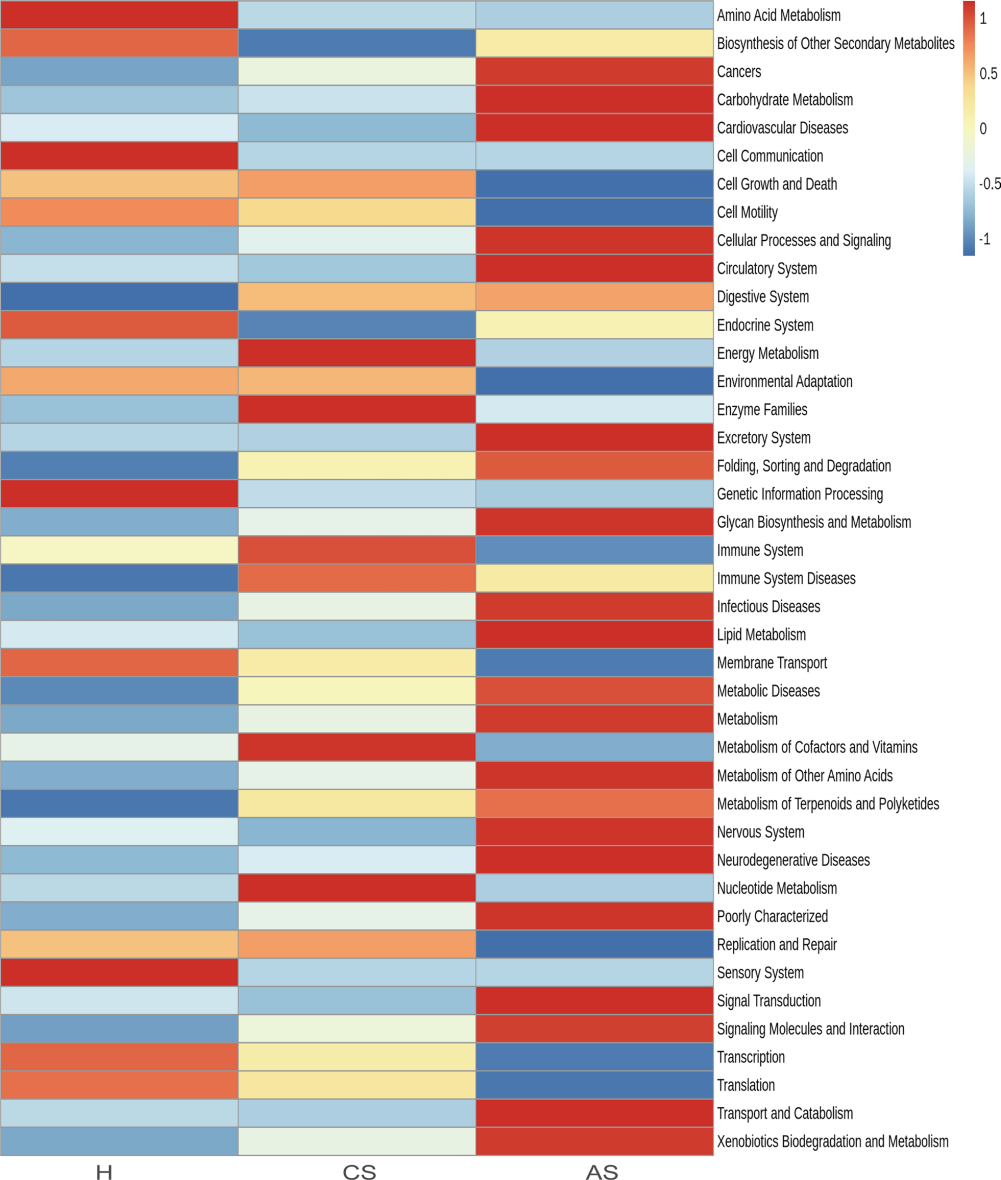
Heatmap showed more comprehensive functional differences among groups

To explore the functional changes of intestinal flora in patients with chronic *S. japonicum* infection and those with advanced *S. japonicum* infection, we used the KEGG database for functional prediction analysis. PCA analysis was undertaken to investigate functional differences among the three groups (Fig. 4A). We performed an LEfSe analysis of the three groups (LDA score > 2). Our results suggested that the functions of the gut microbiota in healthy people were mainly related with transcription and environmental adaptation, while that in patients with chronic *S. japonicum* infection were enriched in immune system diseases, digestive system and cell growth and death. When the disease progressed to an advanced stage, the functional changes were mainly associated with glycan biosynthesis and metabolism, metabolism of other amino acids, neurodegenerative diseases and infectious diseases (Fig. 4B). Heatmap showed more comprehensive functional differences between healthy people and patients with chronic *S. japonicum* infection (Fig. 5).

### Multivariate statistical analysis of metabolites

**Fig. 6:**
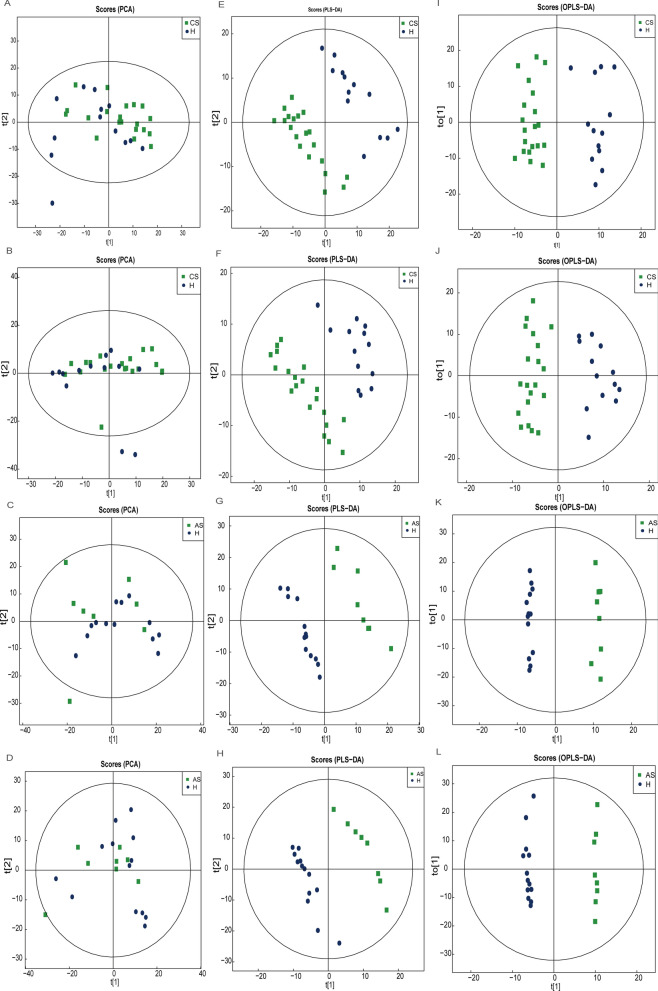
Multivariate statistical analysis of intestinal metabolites in three groups. **A** PCA analysis of chronic group and healthy group in positive electrospray ionization (ESI +) mode. **B** PCA analysis in negative electrospray ionization (ESI-) mode. **C** PCA analysis of advanced group and healthy group in (ESI +) mode. **D** PCA analysis of advanced group and healthy group in (ESI-) mode. **E** PLS-DA analysis of chronic group and healthy group (ESI +) mode. **F** PLS-DA analysis (ESI-) mode. **G** PLS-DA analysis of advanced group and healthy group in (ESI +) mode. **H** PLS-DA analysis of advanced group and healthy group in (ESI-) mode. (I) OPLS-DA analysis of chronic group and healthy group (ESI +) mode. **J** OPLS-DA analysis (ESI-) mode. **K** OPLS-DA analysis of advanced group and healthy group in (ESI +) mode. (L) OPLS-DA analysis of advanced group and healthy group in (ESI-) mode. *H* healthy people, *CS* chronic *Schistosoma japonicum* infection, *AS* advanced *S. japonicum* infection, *PCA* principal component analysis, *PLS-DA* partial least square discriminant analysis, *OPLS-DA* orthogonal partial least squares discriminant analysis. *ESI* electro-spray ionization

To investigate the overall distribution trend and difference of metabolites in three groups, PCA analysis was carried out. QC sample distribution was used for quality inspection, and the results showed that the quality of metabolite detection was accepted (Additional file 1: Fig. S1). We found that the metabolic components of patients with chronic *S. japonicum* infection did not show significant difference compared with healthy people (Fig. 6A, B). However, as the disease progressed to the late stage, metabolites were significantly altered (Fig. 6C, D). To explore the association between patients' metabolites and disease progression, we conducted PLS-DA (Fig. 6E–H) and OPLS-DA analysis (Fig. 6I–L). It showed that the metabolites of patients with *S. japonicum* infection were changed compared with those in healthy people, and the difference gradually expanded as the disease progressed in both ESI modes.

### Identification of differential metabolites

**Fig. 7:**
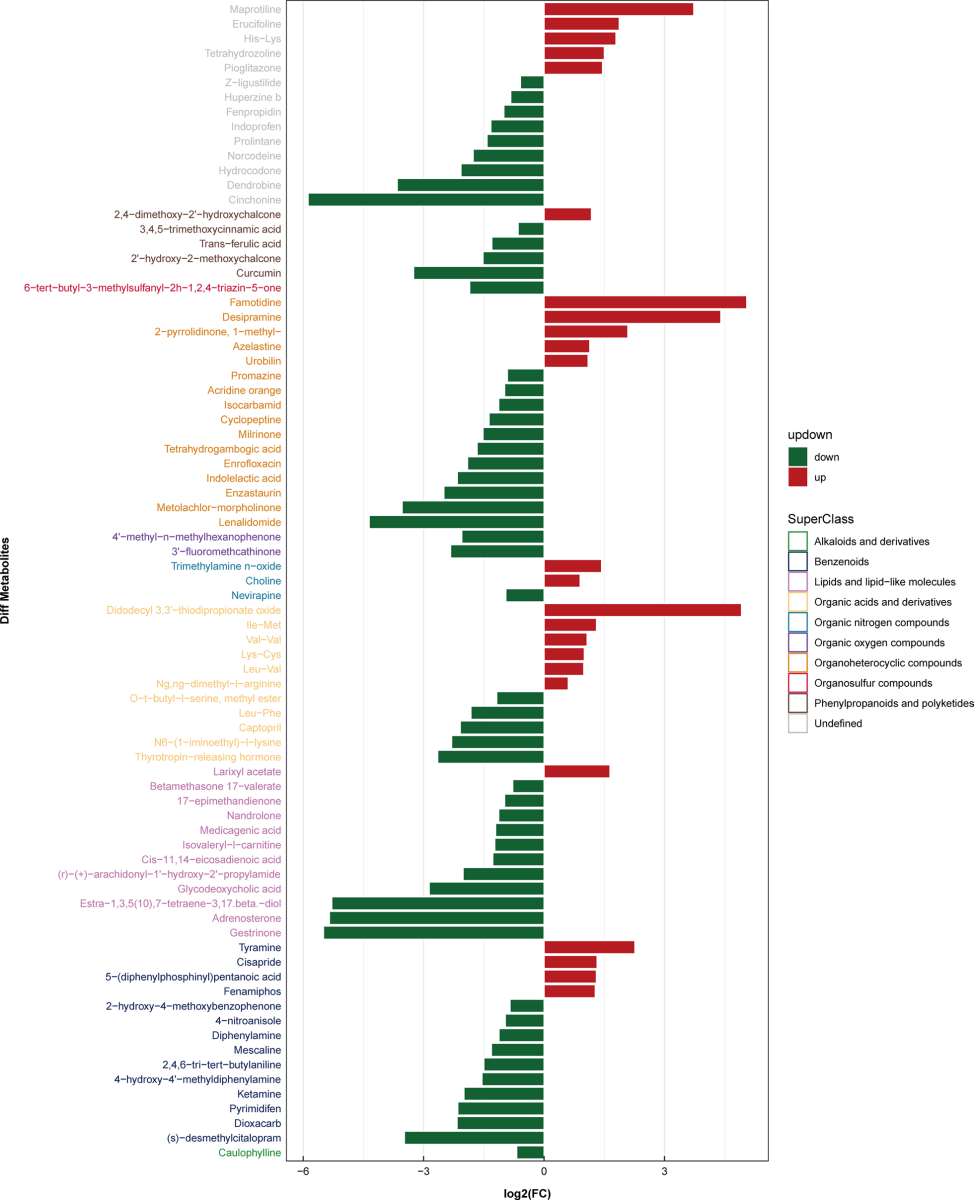
Metabolic changes of patients with chronic *Schistosoma japonicum* infection in ESI + mode

**Fig. 8:**
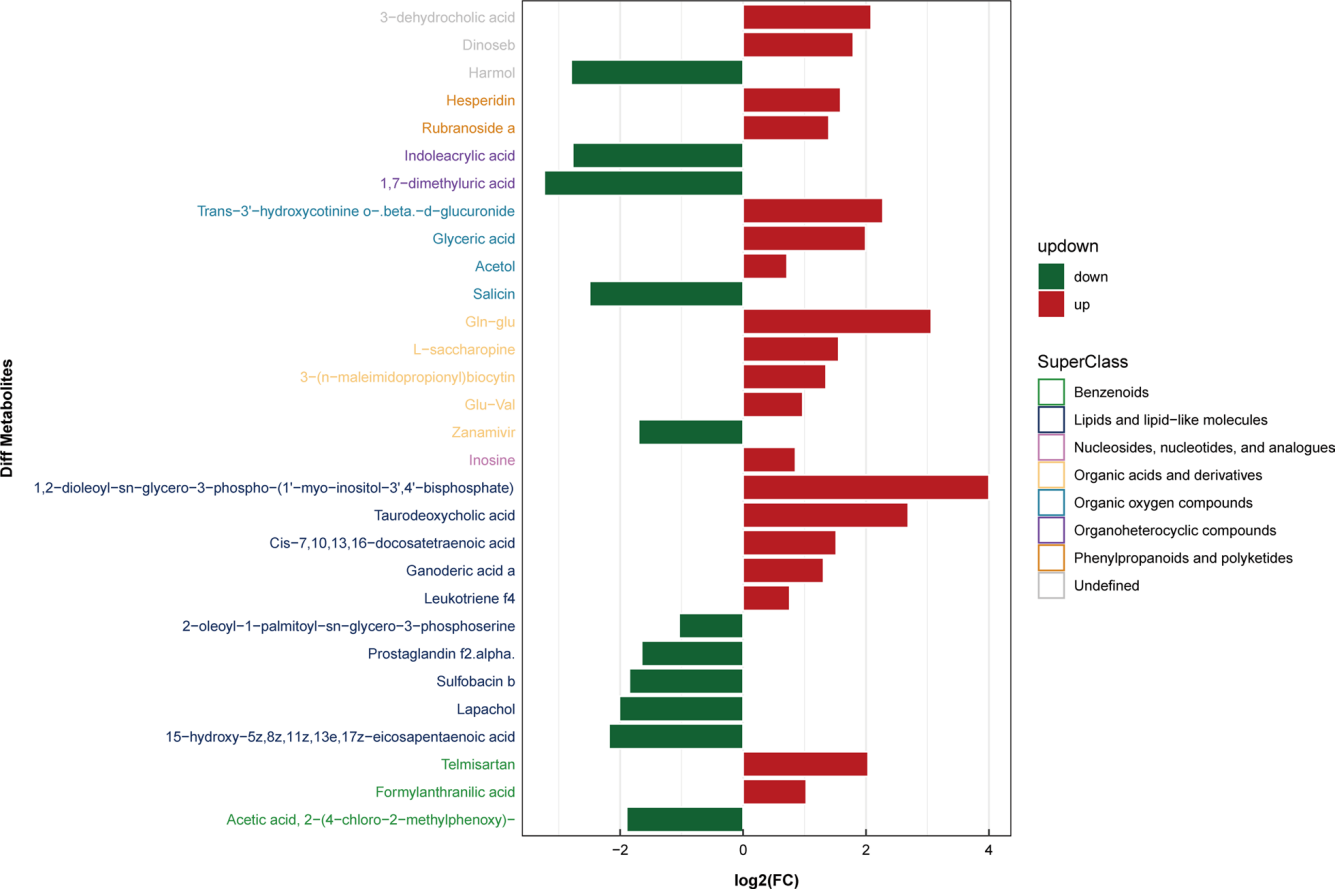
Metabolic changes of patients with chronic *Schistosoma japonicum* infection in ESI- mode

**Fig. 9:**
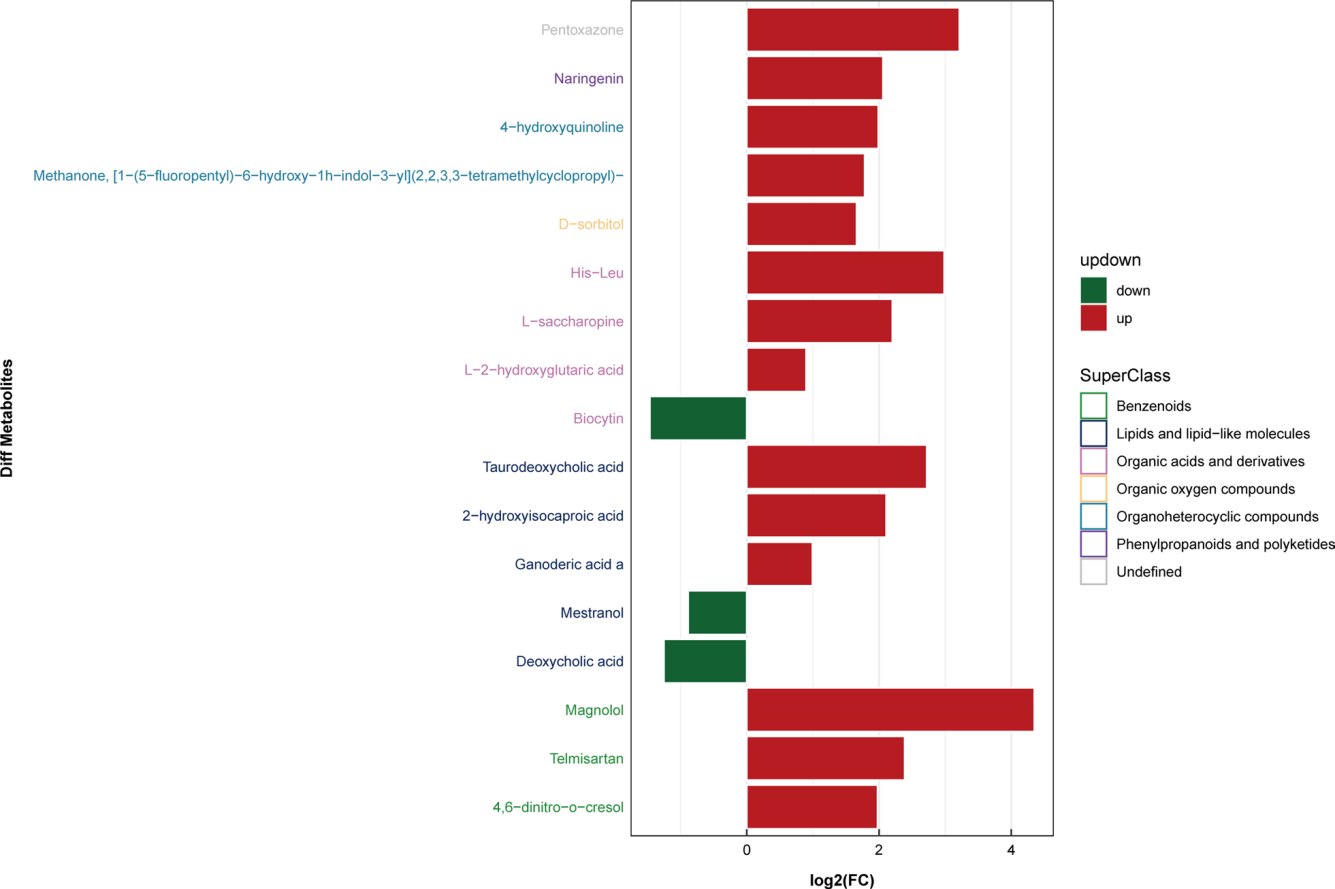
Metabolic changes of patients with advanced *Schistosoma japonicum* infection in ESI- mode

**Fig. 10:**
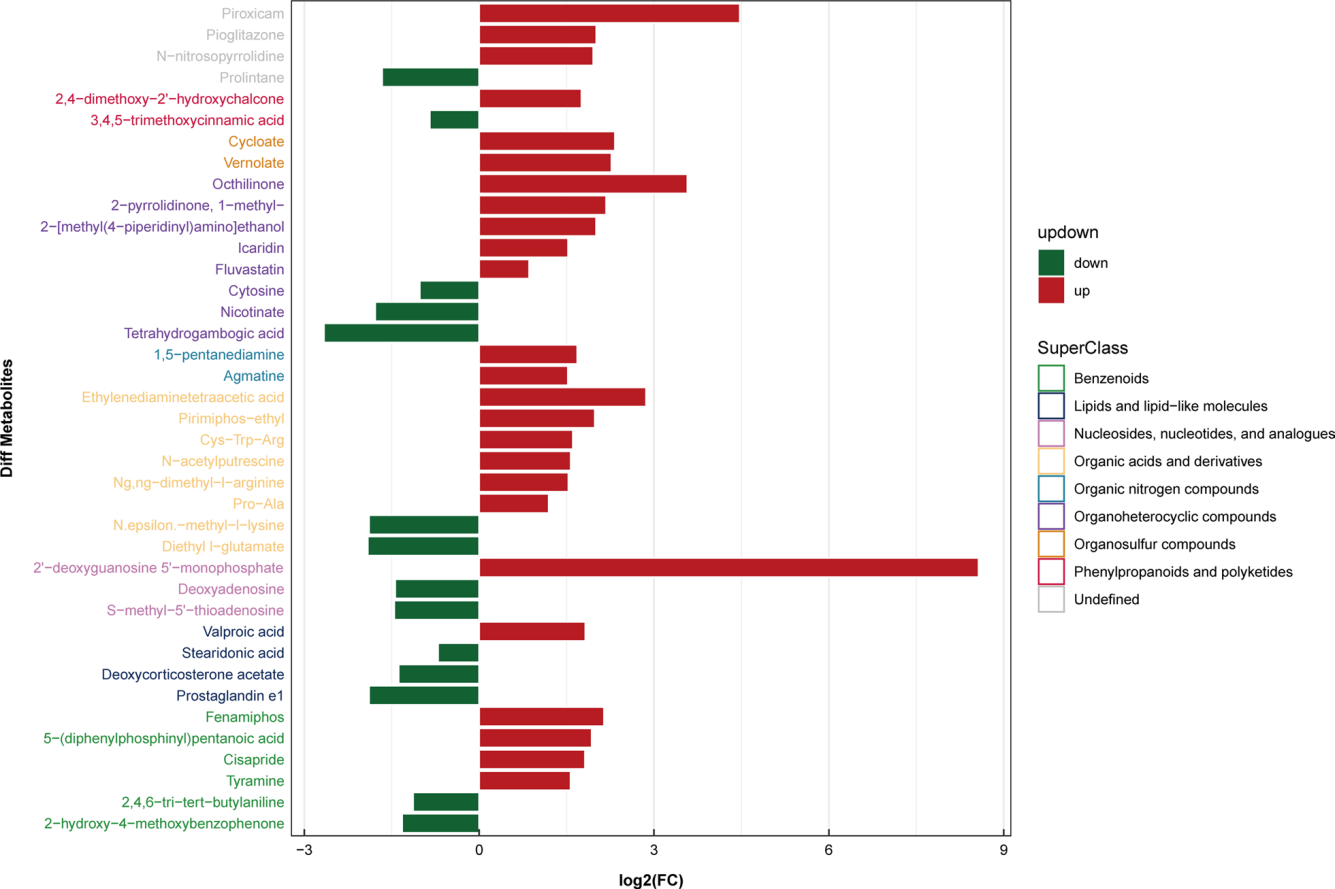
Metabolic changes of patients with advanced *Schistosoma japonicum* infection in ESI + mode

The variable importance for the projection (VIP) is an indicator derived from the OPLS-DA model for evaluating the importance of metabolites. In our study, metabolites with VIP > 1 and *P* value < 0.05 were considered to have significant occurrence in the disease process. The results showed that compared with healthy people, 55 metabolites in patients with chronic *S. japonicum* infection changed significantly (Additional file 2: Table S1), and 39 metabolites changed significantly in patients with advanced *S. japonicum* infection (Additional file 3: Table S2). Among all the metabolites identified, seven changed significantly in both patients with chronic *S. japonicum* infection and patients with advanced *S. japonicum* infection (Additional file 4: Table S3). To reflect the changes of metabolites in patients with *S. japonicum* infection more intuitively, and to compare the internal metabolites of each group, we used a histogram to show the changes of metabolites in patients with chronic *S. japonicum* infection and those with advanced *S. japonicum* infection. Compared with the healthy group, the metabolic changes of patients with *S. japonicum* infection were mainly related to lipids and lipid-like molecules, organo-heterocyclic compounds and benzenoids (Fig. 7), among which the change of lipids and lipid-like molecules ranked first (Fig. 8). The metabolites of lipids and lipid-like molecules in patients with advanced *S. japonicum* infection also changed greatly (Fig. 9). In addition, the metabolic levels of organo-heterocyclic compounds and organic acids and derivatives in patients with advanced *S. japonicum* infection also changed greatly (Fig. 10).

### Correlation between gut microbiota and metabolome

**Fig. 11:**
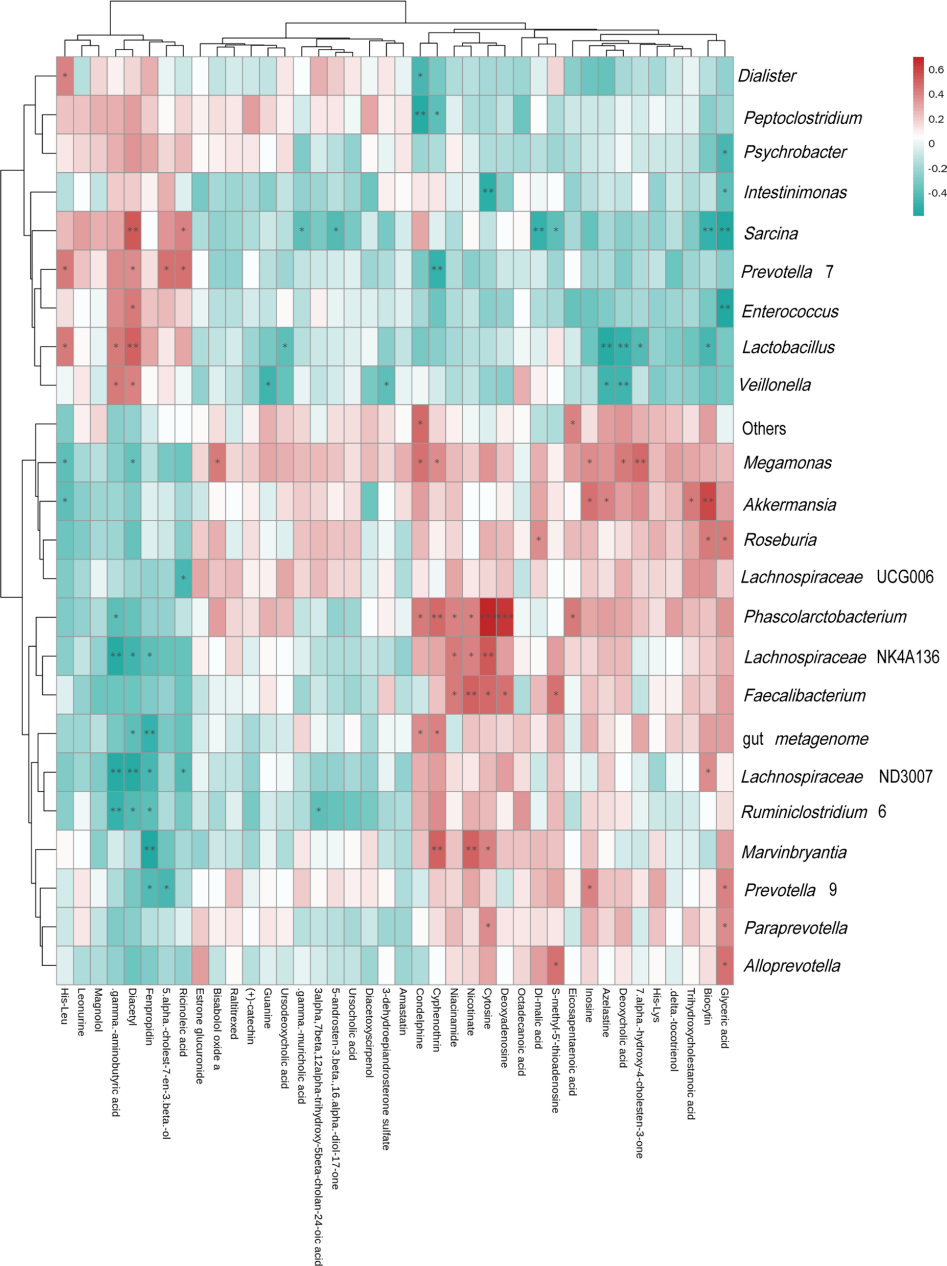
Correlation between gut microbiota and metabolites

Alterations in gut metabolites are closely related to gut microbes. To investigate the correlation between the altered gut microbes and metabolome alteration during the progression of schistosomiasis, we performed a Spearman correlation hierarchical clustering analysis. As presented in hierarchical clustering heatmaps (Fig. 11), both positive and negative correlations between gut microbiome and metabolome alterations were identified. Of note, *Faecalibacterium* was found to be positively correlated with metabolites such as cytosine and nicotinate. Metabolites were also shown to be correlated with various microorganisms, such as glyceric acid, etc. Thus, combined analysis of gut microbiota and metabonomic data suggested that there are interactions between alterations in the gut microbiota and metabolome in the progress of *S. japonicum* infection, which will promote the understanding the mechanism of schistosomiasis.

## Discussion

*Schistosoma japonicum* infection has long been a threat to human health and economy, affecting a wide range of people worldwide [13]. At present, there is no effective diagnostic technology for the distinction between patients with *S. japonicum* infection and patients with advanced *S. japonicum* infection, which affects the treatment decision-making [14]. As a hepatic-intestinal parasitic disease, schistosomiasis is closely associated with intestinal microbes. In a study of the microbiota of patients with acute *S. japonicum* infection, *Bacteroides* enterotype was found [15]. Our previous study also showed that dysbiosis of gut microbiota occurred in patients with chronic or advanced *S. japonicum* infection [8]. In our study, there was gut bacterial dysbiosis in patients with *S. japonicum* infection. Results suggested that the intestinal microbiota community structure changed in patients with chronic and advanced *S. japonicum* infection. The gut microbiota of patients with *S. japonicum* infection were dominated by *Firmicutes, Bacteroides* and *Proteobacteria*. As the disease progressed, *Firmicutes* decreased while *Proteobacteria* increased, consistent with observations in previous studies [16]. To explore the changes of gut microbial diversity in patients with *S. japonicum* infection, we compared the diversity of three groups. The results showed that patients with chronic and advanced *S. japonicum* infection had lower alpha and beta diversity compared with healthy people, but there was no statistical difference in the diversity of gut microbiota between patients with chronic and advanced *S. japonicum* infection. The alpha diversity of the gut microbiota was reduced in patients with *S. japonicum* infection, consistent with previous studies [16], but there is currently controversy over the changes in the beta diversity of the gut microbiota after schistosomiasis infection [17]. After analyzing the gut microbiota at the genus level, we found a group of microorganisms that were characteristically enriched in patients with chronic and advanced *S. japonicum* infection. *Faecalibacterium*, which was abundantly enriched in healthy people, decreased continuously after infection with *S. japonicum*. Previous studies found *Faecalibacterium* was reduced in the gut of NAFLD patients [18]. It was also reported in the study of hepatitis C [19], indicating a close relationship between *Faecalibacterium* and liver-related diseases. *Faecalibacterium* in patients with *S. japonicum* infection continually decreased, suggesting liver damage in patients with *S. japonicum* infection. *Prevotellaceae* helps break down proteins and carbohydrates and is considered as a probiotic, reduced in various diseases. However, recent studies have linked increased *Prevotella* to T helper type 17 (Th17)-mediated mucosal inflammation [20]. Interestingly, the increase of *Prevotella* was reported in both colitis mice [21] and *S. japonicum*-infected mice [22]. We found an increase in *Prevotella* in patients with chronic *S. japonicum* infection, indicating *Schistosoma*-associated inflammation of the intestinal mucosa in patients with *S. japonicum* infection. When in advanced stage, the reduction of *Prevotella* content may impair the patient's digestion of protein and carbohydrates, resulting in frailty.

The analysis of the community structure, diversity and characteristic species of the three groups revealed the alterations in the gut microbiota from chronic to advanced stage. Subsequent studies of functional and metabolic alterations could help us better understand how patients were affected by *S. japonicum* in the development of disease. The results of functional analysis based on the KEGG database suggested that the functional changes in patients with chronic *S. japonicum* infection were mainly related to diseases of the immune and digestive systems. This result indicated the pathological process of chronic schistosomiasis because, in the chronic stage, granulomas and eggs activated the patient's immune system, leaving the patient's immune system in an abnormal state for a long time[23]. In addition, at this stage, liver and intestinal damage and tissue fibrosis occurred [24]. The patient's digestive function was weakened. As the disease progressed to the advanced stage, the patient's liver fibrosis was severe and uncompensated. The patient's metabolic function was abnormal. In addition, patients with abnormal energy metabolism often developed weak immunity, which increased the risk of infectious diseases.

There have been animal experiments to study the changes of organism metabolism after *S. japonicum* infection [25]. Our results showed that the metabonomics of humans with *S. japonicum* infection changed, and this difference contributed to the progress of the disease, consistent with previous animal studies[7].

The alteration in metabolism in patients with *S. japonicum* infection remained unclear. Our results showed that lipids and lipid-like molecules and organo-heterocyclic compounds were the main metabolites of patients with *S. japonicum* infection. Lipids are important compounds that constitute many biofilms [26], and they also play an important role in intracellular signal transmission [27]. In patients with chronic schistosomiasis, 2-oleoyl-1-palmitoyl-sn-glycero-3-phosphoserine and Cis-7,10,13,16-docosatetraenoic acid are two important lipids. 2-Oleoyl-1-palmitoyl-sn-glycero-3-phosphoserine is a kind of phosphatidylserine, which is an important component of the eukaryotic cell membrane [28]. When exposed to the cell surface, phosphatidylserine can promote the clearance of apoptotic cells [29]. In addition, some studies demonstrated that it can protect the body and liver cells by regulating inflammation [34, 35]. In our study, phosphatidylserine in patients with chronic *S. japonicum* infection was lower than that in normal people, which may reflect the decreased liver inflammation inhibition ability and insufficient clearance of apoptotic liver cells in chronic schistosomiasis patients. Cis-7,10,13,16-docosatetraenoic acid is another important lipid that was upregulated in patients with chronic *S. japonicum* infection. As an endogenous polyunsaturated free fatty acid, it is increased in patients with nonalcoholic fatty liver disease (NAFLD) and NAFLD model mice, closely related to oxidative stress and cell death [30]. Thus, Cis-7,10,13,16-docosatetraenoic acid may have a potential effect on liver cell injury and liver inflammation in patients with *S. japonicum* infection. In addition to lipids, we found significant changes in indole acrylic acid in patients with chronic *S. japonicum* infection. As a kind of organo-heterocyclic compound, previous studies suggested that it could maintain a normal intestinal barrier [31]. Our study found that it decreases significantly in patients with chronic *S. japonicum* infection, which may cause intestinal barrier damage and liver inflammation in patients with chronic *S. japonicum* infection. An important lipid that changes in patients with advanced *S. japonicum* infection is prostaglandin e1(PGE1), which is a subclass of lipid mediators called eicosanoids [32]. The protective effect of PGE1 has been proved in an experimental animal model of liver injury [33] and patients with fulminant viral hepatitis[34]. Compared with healthy people, the content of PGE1 in patients with advanced *S. japonicum* infection was lower, indicating severe liver injury and inflammation. Nicotinic acid is a kind of organo-heterocyclic compound changed in patients with advanced *S. japonicum* infection, which plays an important role in energy metabolism[35] and DNA repair of cells[36]. The liver can synthesize nicotinic acid from tryptophan. In our study, we found that nicotinic acid in patients with advanced *S. japonicum* infection decreased significantly, which suggested that the synthesis function of patients with advanced *S. japonicum* infection decreased and the energy metabolism was disordered because of severe liver damage. His-Leu is a carboxyl-terminal dipeptide of Ang I, which is cleaved by ace to produce Ang II. The gene expression of Ang II was different between early and late fibrosis patients with NASH [37], and the high level of Ang II was related to severe liver fibrosis [38]. In our study, we found that the patients with advanced *S. japonicum* infection had a high level of His-Leu, which indicated a high level of Ang II and severe fibrosis. In addition, the level of organic acids and derivatives in patients with advanced *S. japonicum* infection also changed significantly. Organic acid is the intermediate product of amino acid catabolism, and its abnormality often reflects the metabolic disorder of the body [39]. Because the liver of patients with advanced *S. japonicum* infection is seriously damaged and uncompensated, it often shows metabolic disorder [40]. Therefore, organic acid has the potential to be a metabolic marker for distinguishing patients with chronic *S. japonicum* infection from patients with advanced *S. japonicum* infection and evaluating their liver metabolic function.

In addition to the metabolites mentioned above, our research also discovered a number of metabolites changed in both patients with chronic *S. japonicum* infection and those with advanced *S. japonicum* infection. 3,4,5-Trimethoxycinnamic acid decreased in patients with chronic *S. japonicum* infection and further decreased in patients with advanced *S. japonicum* infection. It can block the adhesion of neutrophils to endothelial cells by inhibiting the expression of cell adhesion molecules [41]. The adhesion of leukocytes to endothelial cells is a basic event in many early inflammatory stages [42], and the decrease of metabolite level accords with the inflammatory changes in patients with *S. japonicum* infection. Ng, ng-dimethyl-l-arginine is a metabolic by-product of continuous protein modification in the cytoplasm of all human cells. It can interfere with the production of nitric oxide by l-arginine [43]. Previous studies found that the reduction of endogenous nitric oxide (NO) can lead to liver injury [44]. Our study found that this metabolite increased with the progress of *S. japonicum* infection, and it may aggravate liver injury by reducing the synthesis of nitric oxide in their bodies. Tyramine is an important monoamine compound [45]. Monoamine oxidase (MAO) is an enzyme reflecting liver fibrosis, which exists in mitochondria of liver, kidney and other tissues [46]. The level of tyramine in patients with chronic *S. japonicum* infection increased, but it decreased in advanced stage. This was related to the aggravation of liver fibrosis and increase of MAO activity in patients with advanced *S. japonicum* infection, which leads to the increase of liver metabolism level of tyramine and leads to the decrease of tyramine level. Therefore, tyramine seemed to have potential to distinguish patients with chronic *S. japonicum* infection from those with advanced *S. japonicum* infection.

Although our study revealed the alterations in gut microbiota and metabolomics in patients with *S. japonicum* infection in different stages, absence of patients with acute *S. japonicum* infection limited the understanding of the potential role of gut microbiota and metabolome in acute phase. Because of schistosomiasis control in China, prevalence of acute *S. japonicum* infection is quite low[47], and no patient in acute phase was found during the course of our research. The sample size of this study is also low, and more studies with larger sample size are needed. The mechanism and roles of gut microbiota and metabolomics in *S. japonicum* infection require further research.

## Conclusions

This study revealed alterations in gut microbiota and metabolites in patients in different stages of schistosomiasis. We found that the alpha and beta diversity decreased when patients were infected with *S. japonicum* infection and alteration in gut microbiota occurred when patients transited from chronic to advanced *S. japonicum* infection. With LEfSe analysis, we found several bacteria that have potential to act as biomarkers for diagnosis of patients with chronic and advanced *S. japonicum* infection. Furthermore, functional analysis makes it clear that the functional changes of gut microbiota in patients with chronic *S. japonicum* infection were mainly focused on inflammation and digestive diseases like cholecystitis, gastric ulcer, etc., while that in patients with advanced *S. japonicum* infection was mainly associated with metabolic abnormalities. Our results showed that the metabolics of patients with *S. japonicum* infection changed as the disease progressed, and this was associated with disease progression. In addition, the most changed metabolites in patients with *S. japonicum* infection were lipids and lipid-like molecules and organo-heterocyclic compounds. These metabolites were closely related. The intestinal flora and metabolism have been mutually proved in this study and were closely correlated with the clinical manifestations and disease progression of schistosomiasis caused by *S. japonicum*. This study on intestinal microbes and metabolism will promote further understanding of the pathology and disease development caused by *S. japonicum* and contribute to finding new targets for the diagnosis and treatment of the parasitic disease. However, further validation in clinic is needed. In addition, the causal relationship between them and the underlying mechanism remained unclear, suggesting further study is required.

### Supplementary Information

**Additional file 1: Fig. S1. **PCA analysis containing quality control. H, healthy people; CS, chronic *Schistosoma japonicum* infection; AS, advanced *S. japonicum* infection; PCA, principal component analysis; QC, quality control.

**Additional file 2: Table S1.** Metabolites in patients with chronic *Schistosoma japonicum* infection changed significantly.

**Additional file 3: Table S2.** Metabolites in patients with advanced *Schistosoma japonicum* infection changed significantly.

**Additional file 4: Table S3.** Metabolites changed significantly in both patients with chronic and with advanced *Schistosoma japonicum* infection

## Data Availability

The sequencing data have been deposited in the NCBI Sequence Read Archive under the SRP459542.

## Abbreviations

ANOSIM: Analysis of similarities
16S r RNA: 16S ribosomal ribonucleic acid
DNA: Deoxyribonucleic acid
ESI: Electro-spray ionization
KEGG: Kyoto Encyclopedia of Genes and Genomes
LDA: Linear discriminant analysis
LEfSe: Linear discriminant analysis effect size
NMDS: Non-metric multidimensional scaling
OPLS-DA: Orthogonal partial least square discriminant analysis
PCR: Polymerase chain reaction
PCA: Principal component analysis
PCoA: Principal coordinate analysis
PLS-DA: Partial least square discriminant analysis
*S. japonicum*: *Schistosoma japonicum*
