# Supplementary figures and images for: Alterations in gut microbiome and metabolite profile of patients with Schistosoma japonicum infection
Source: Parasit Vectors. 2023 Oct 5;16:346. doi: 10.1186/s13071-023-05970-3 (PMC10552355; doi:10.1186/s13071-023-05970-3)

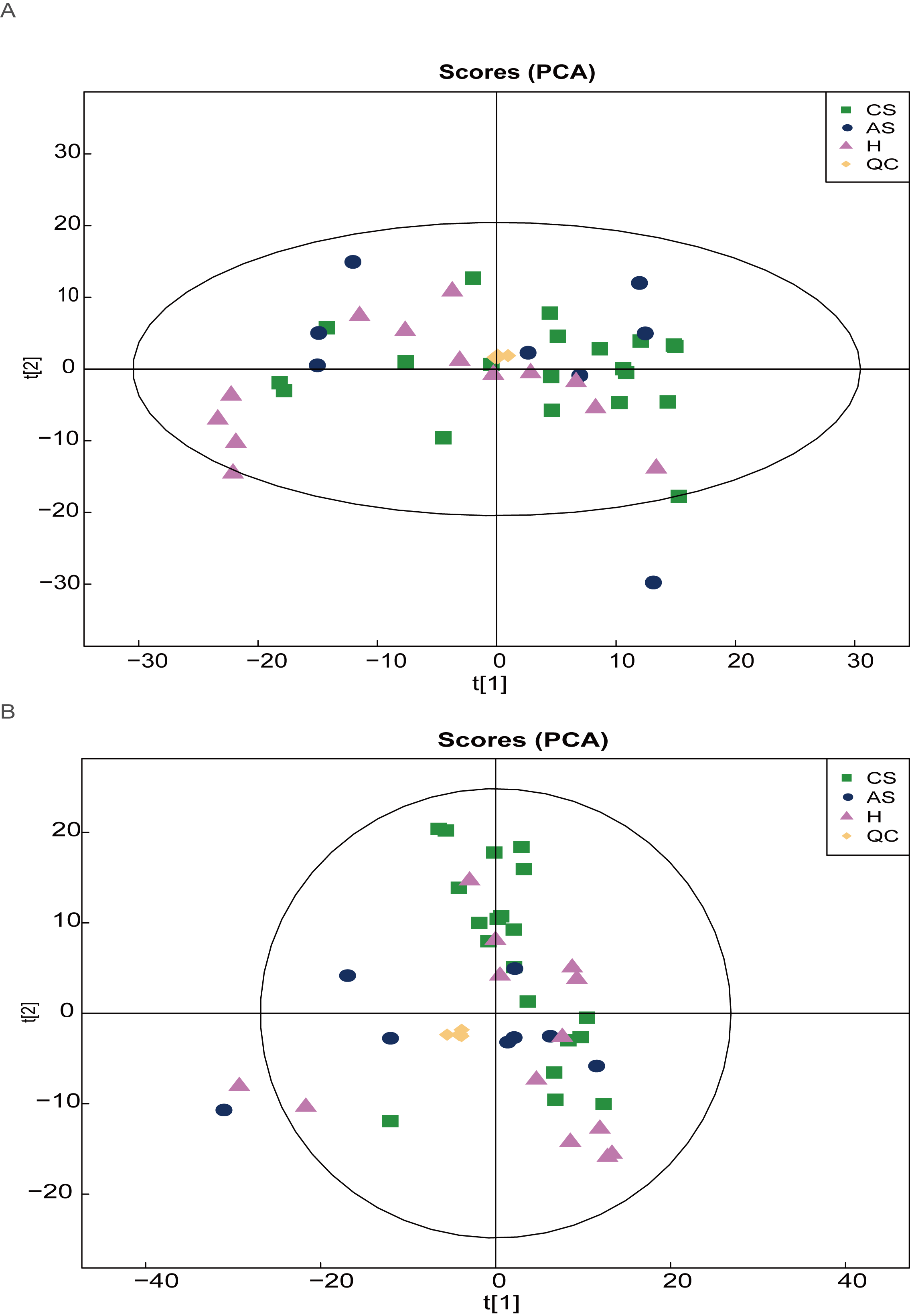

Supplement: Supplementary file 1 — Additional file 1: Fig. S1. PCA analysis containing quality control. H, healthy people; CS, chronic Schistosoma japonicum infection; AS, advanced S. japonicum infection; PCA, principal component analysis; QC, quality control. [file 13071_2023_5970_MOESM1_ESM.tif]
